# Double jeopardy: long QT3 and Brugada syndromes

**DOI:** 10.1002/ccr3.1064

**Published:** 2017-06-30

**Authors:** Amneet Sandhu, Ryan T. Borne, Chandara Mam, T. Jared Bunch, Ryan G. Aleong

**Affiliations:** ^1^ Division of Cardiovascular Medicine Department of Internal Medicine The University of Colorado Aurora Colorado; ^2^ Calmette Hospital Phnom Penh Cambodia; ^3^ Intermountain Medical Center Heart Institute Salt Lake City UT, USA

**Keywords:** Brugada syndrome, genetic channelopathy, long QT syndrome, sudden cardiac death

## Abstract

Mutations in the SCN5A gene are linked to both the long QT syndrome 3 and Brugada syndrome with few reports describing an overlapping phenotype. We present a unique case and discuss clinical considerations of a patient concurrently exhibiting such conditions with genetic analysis confirming an SCN5A mutation.

## Introduction

Brugada syndrome is a genetic disorder resulting in abnormal sodium ion channel function conferring an increased risk of ventricular arrhythmias, syncope and sudden cardiac death. Although rare, this increasingly recognized condition affects 4 of 1000 people in the United States 1. Inherited as an autosomal dominant trait with incomplete penetrance, mutations in the SCN5A gene represent the most common genotype.

However, mutations in the SCN5A gene can be present in both the long QT syndrome 3 (LQT3) in addition to Brugada syndrome 2, 3. A prior report of a particular SCN5A mutation has described phenotypic long QT and Brugada pattern ST segment elevation in the anterior leads further obscuring the distinction between these syndromes 4. Despite growing genetic overlap between these syndromes and differing management strategies, there have been few reports describing phenotypic presentations consistent with both syndromes and clinical experience with overlap of these disorders. We report a case of a young patient who exhibited phenotypic characteristics of both syndromes including type 1 Brugada pattern with provocation and baseline prolonged QT with genetic analysis confirming a mutation in the SCN5A gene associated with both LQT3 and Brugada syndromes. Importantly, we discuss clinical considerations given differing management approaches.

## Case

A 38‐year‐old male with a significant family history of Brugada syndrome presented for clinical evaluation at an outside facility. The patient noted that his father carries the SCN5A mutation despite an unremarkable EKG. In addition, he has two paternal cousins with Brugada syndrome, one of whom suffered from sudden cardiac arrest at age 40 and the other received an implantable cardioverter defibrillator (ICD). The patient's paternal uncle was positive for the SCN5A mutation and experienced sudden cardiac death at age 60. Lastly, the patient's daughter was noted to have a spontaneous type 1 Brugada pattern on surface ECG in addition to a prolonged QT interval and is currently undergoing genetic testing.

**Figure 1:**
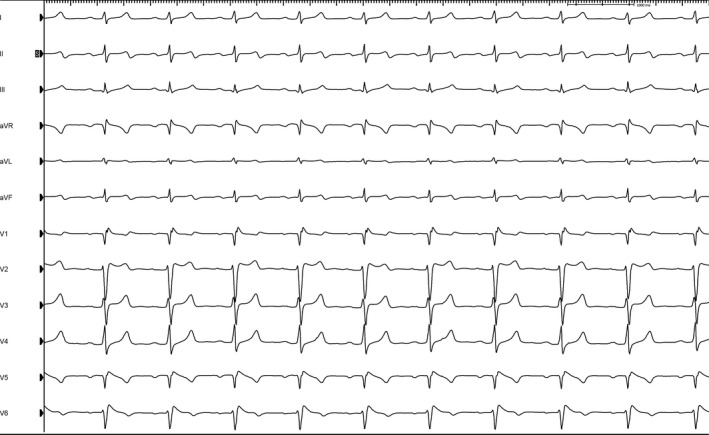
Electrocardiogram of our patient showing concern for type 2 Brugada pattern, baseline bradycardia, and long QT interval.

Given an impressive family history, our patient sought evaluation from a local cardiologist for dizziness with exertion that he began to experience 6 months prior. He denied syncope but noted palpitations. At baseline, there were findings suggestive of type 2 Brugada ECG pattern (Fig. 1) with J point elevation and saddle‐back ST segments in the anterior precordial leads. Workup with a treadmill electrocardiogram (ECG) stress testing was unremarkable. Mobile telemetry showed premature ventricular contractions (PVCs) originating from the right ventricular outflow tract (RVOT) associated with palpitations and chest pain. Genetic analysis revealed the patient was positive for the c.5350G>A; p.Glu1784Lys mutation in the SCN5A gene. He was referred to the electrophysiology department at our institution for further workup.

On evaluation, the patient corroborated symptoms of dizziness and chest pain associated with RVOT PVCs. His cardiovascular medications persisted despite starting metoprolol 25 mg extended‐release. Physical examination was unremarkable with normal heart sounds, no cardiovascular murmurs or signs of congestive heart failure. An outside transthoracic echocardiography showed normal right and left ventricular systolic function with no significant valvular pathology. On thorough discussion with the patient regarding his family history, ECG, clinical symptoms and concern for LQT3 and Brugada syndrome expression given the genetic analysis, we reviewed the role of provocative testing in the electrophysiology (EP) laboratory to help define a diagnosis of Brugada syndrome. In addition, we discussed the role programmed ventricular stimulation for risk stratification of sudden cardiac death as induction of ventricular arrhythmias has been shown to increase the risk of sudden death or appropriate ICD therapies twofold to threefold 5.

**Figure 2:**
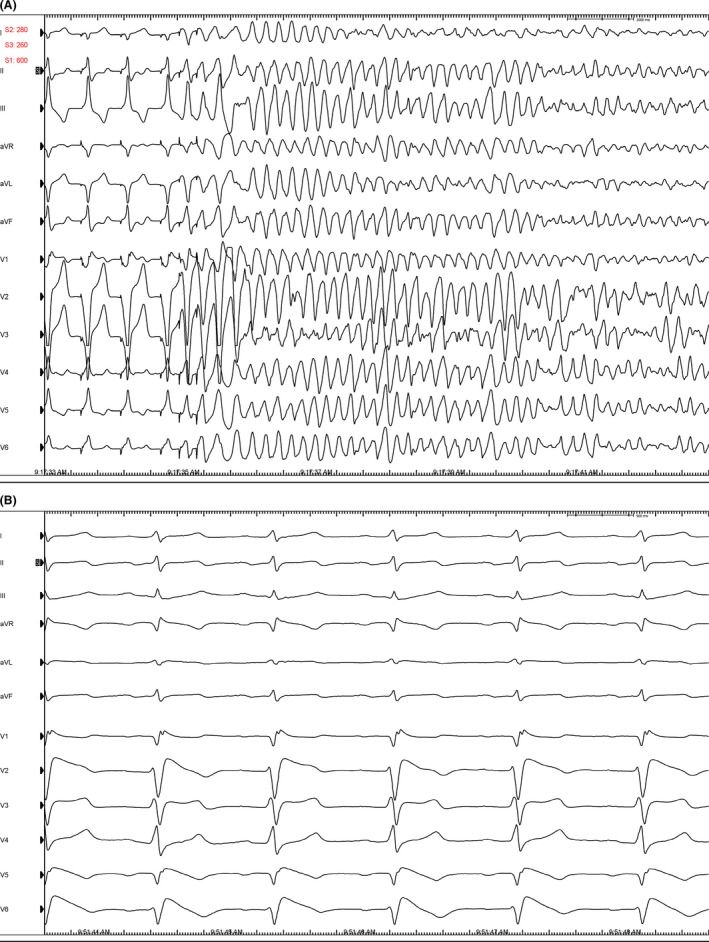
(A) VF induction with ventricular double extrastimuli. (B) CardioLab tracings showing provocation of type 1 Brugada pattern with procainamide infusion.

During EP study, the patient's baseline cardiac intervals were within normal limits other than an absolute QT interval of 501 milliseconds at a heart rate of 61 bpm, resulting in a corrected QTc of 500 milliseconds. This was followed by programmed ventricular stimulation in the right ventricular apex. Polymorphic ventricular tachycardia was induced with double extrastimulation that degenerated into ventricular fibrillation, consistent with a higher risk phenotype (Fig. 2 – panel A) 5. Procainamide challenge in the EP laboratory revealed a type 1 Brugada pattern (Fig. 2 – panel B). Given results from EP study, we elected to implant a subcutaneous ICD. The patient tolerated the procedure well and has not experienced device therapies to date.

## Discussion

To date, there have been few reports describing clinical experiences of overlap between LQT3 and Brugada syndrome. In the National Heart Lung Blood Institute (NHLBI) Exome Sequencing, the c.5350G>A; p.Glu1784Lys missense mutation in the SCN5A gene has been implicated in the pathogenesis of both LQT3 and Brugada syndrome 6, 7. With known links in the genetic basis between these two conditions 4, this case expands this genotypic link and further illustrates phenotypes representing an overlap between LQT3 and Brugada syndromes. Importantly, it emphasizes the possibility of coexisting manifestations of these conditions given differing management approaches.

Prior studies have shown genetic overlap between frequently reported mutations such as SCN5A‐E1784K leading to manifestation as LQT3 or Brugada phenotypes 8. Preceding this, Priori et al. 7 had shown induction of the type 1 Brugada pattern in a subset of patients with LQT3. The confirmation of genotype–phenotype overlap between LQT3 and Brugada syndrome with this mutation suggests an important connection between these conditions that perhaps lie along a single spectrum of disease. The case presented builds on our current understanding of these disorders in illustrating a novel genetic mutation further corroborating a genotypic–phenotypic link underlying these heritable conditions.

Despite common underlying genetic pathology between LQT3 and Brugada syndrome, current suggested management strategies differ and may provide conflicting recommendations. The 2013 Heart Rhythm Society (HRS) expert consensus recommendations discuss the role of avoiding trigger medications, treatment of fever, the utility of ICDs, and anti‐arrhythmic (e.g., quinidine) medications as central therapies in the management of Brugada syndrome. In contrast, the recommendations for long QT syndrome focus on use of beta‐blockers and discuss the role for ICDs in those who have suffered from cardiac arrest or syncope. In specific, they provide a Class IIa recommendation or use of sodium channel blockers (e.g. flecainide) to potentiate QT shortening in LQT3 9. Yet, despite QT shortening with use of flecainide in LQT3, prior data have shown induction of type 1 Brugada ECG pattern in a significant number of patients 7. These data raise concern regarding the use of sodium channel blockers in certain patients, such as ours, that exhibit coexisting phenotypic characteristics of LQT and Brugada syndromes.

In the case of our patient, we had a thorough discussion around concern for concurrent, phenotypic exhibition of LQT3 and Brugada syndrome given resting QT interval, induction of type 1 Brugada syndrome with provocation and genetic testing. Given strong family history of positive genetic testing and sudden cardiac death, it is possible that his family members share a similar phenotype. We discussed the role of medicines for LQT3 and Brugada syndrome, the limited data evaluating sodium channel blockers, and data underlying the role of electrophysiological testing for each inherited arrhythmia syndrome. In our case, the patient opted to undergo electrophysiological testing with easily inducible type 1 Brugada pattern in addition to ventricular fibrillation with double extrastimuli programmed ventricular stimulation. Recent data further elucidated risk stratification in patients with Brugada syndrome showing that induction of ventricular tachyarrhythmias, particularly with ventricular single or double extrastimuli, is a marker of increased risk for sudden cardiac death 5. Consistent with guideline recommendations 10, we asked he avoid “high intensity” activities and engage in modest, monitored exercise. On short‐term follow‐up after uncomplicated ICD implantation, he reports clinical stability without symptoms.

Importantly, this case brings to light the consideration of genetic testing in inherited arrhythmic syndromes. Our patient carried a genetic mutation with an autosomal dominant inheritance pattern. He has children and siblings placing importance on identification of this mutation for counseling of family members. Current HRS guidelines provide a class I recommendation for genetic testing given strong suspicion of LQT syndrome and class IIa recommendation for genetic testing in evaluation of Brugada syndrome 11. In addition, recent work has shown value of genetic testing in Brugada syndrome with future implication on management strategies 12. In practice, genetic testing often is not undertaken due to limited resources, cost or unavailability of testing. For our patient, we recommended prompt genetic testing of his children and family members as results may identify those with the mutation whom are at potential future risk and influence future management decisions. In addition, mutation detection is vital in understanding the variable penetrance of rare genetic disorders.

In conclusion, we describe a unique case with concurrent phenotypic exhibition of LQT3 and Brugada syndromes with a common, underlying genetic mutation in the SCN5A gene. This adds to the growing understanding and genotypic link between these conditions with emerging discoveries in variable penetrance. Importantly, management strategies between these conditions differ and use of sodium channel blockers in treatment of LQT3 may unmask Brugada syndrome. Clinicians should remain aware of genotypic link between LQT3 and Brugada syndrome, recognize the potential for phenotypes consistent with both syndrome, and individualize therapies after thorough discussion with patients.

## Authorship

AS (primary author): involved in initial draft of the manuscript and revised the manuscript. RTB: involved in initial draft of the manuscript and revised the manuscript. CM: involved in initial draft of the manuscript. TJB: revised the manuscript. RGA (senior author): involved in initial draft of the manuscript and revised the manuscript.

## Conflict of Interest

The authors have no conflict of interests to report.
